# Systems approach to design multi-epitopic peptide vaccine candidate against fowl adenovirus structural proteins for *Gallus gallus domesticus*

**DOI:** 10.3389/fcimb.2024.1351303

**Published:** 2024-05-28

**Authors:** Susithra Priyadarhni Mugunthan, Divyadharshini Venkatesan, Chandramohan Govindasamy, Dhivya Selvaraj, Mani Chandra Harish

**Affiliations:** ^1^ Department of Biotechnology, Thiruvalluvar University, Vellore, Tamil Nadu, India; ^2^ Department of Community Health Sciences, College of Applied Medical Sciences, King Saud University, Riyadh, Saudi Arabia; ^3^ Artificial Intelligence Laboratory, School of Computer Information and Communication Engineering, Kunsan National University, Gunsan, Republic of Korea

**Keywords:** fowl adenovirus, *in silico*, multi-epitopic vaccine, avian disease, penton base, hexon, fiber protein

## Abstract

**Introduction:**

Fowl adenovirus (FAdV) is a significant pathogen in poultry, causing various diseases such as hepatitis-hydropericardium, inclusion body hepatitis, and gizzard erosion. Different serotypes of FAdV are associated with specific conditions, highlighting the need for targeted prevention strategies. Given the rising prevalence of FAdV-related diseases globally, effective vaccination and biosecurity measures are crucial. In this study, we explore the potential of structural proteins to design a multi-epitope vaccine targeting FAdV.

**Methods:**

We employed an *in silico* approach to design the multi-epitope vaccine. Essential viral structural proteins, including hexon, penton, and fiber protein, were selected as vaccine targets. T-cell and B-cell epitopes binding to MHC-I and MHC-II molecules were predicted using computational methods. Molecular docking studies were conducted to validate the interaction of the multi-epitope vaccine candidate with chicken Toll-like receptors 2 and 5.

**Results:**

Our *in silico* methodology successfully identified potential T-cell and B-cell epitopes within the selected viral structural proteins. Molecular docking studies revealed strong interactions between the multi-epitope vaccine candidate and chicken Toll-like receptors 2 and 5, indicating the structural integrity and immunogenic potential of the designed vaccine.

**Discussion:**

The designed multi-epitope vaccine presents a promising approach for combating FAdV infections in chickens. By targeting essential viral structural proteins, the vaccine is expected to induce a robust immunological response. The *in silico* methodology utilized in this study provides a rapid and cost-effective means of vaccine design, offering insights into potential vaccine candidates before experimental validation. Future studies should focus on *in vitro* and *in vivo* evaluations to further assess the efficacy and safety of the proposed vaccine.

## 1 Introduction

**Figure 1:**
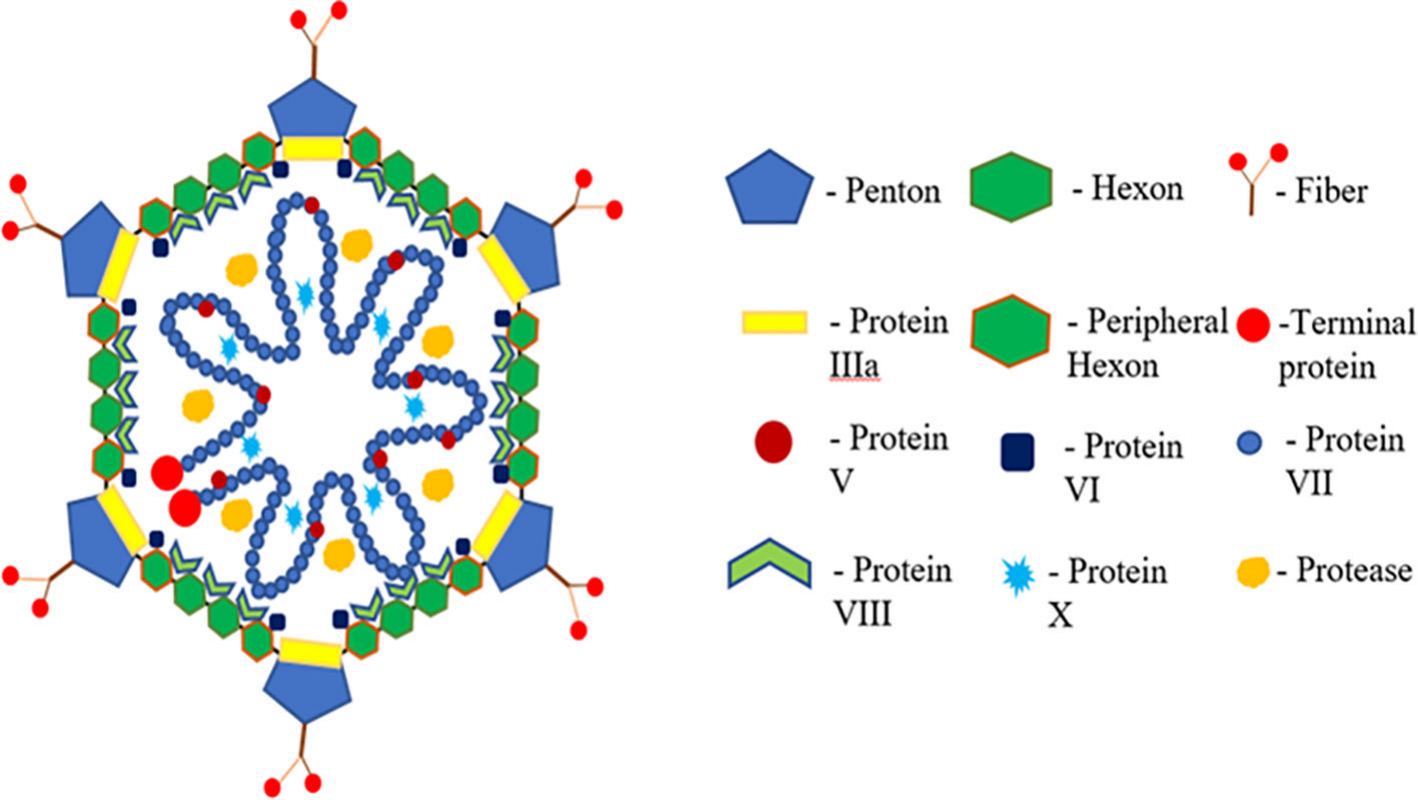
Structure of fowl adenovirus showing structural and functional proteins.

Adenovirus constitutes a vast and diverse viral family, recognized as a significant contributor to diseases in chordates, including avian and mammalian species (Wigand et al., 1982). According to the International Committee on Taxonomy of Viruses (ICTV), Adenoviridae are classified into five genera: Mastadenovirus, Aviadenovirus, Siadenovirus, Atadenovirus, and Ichtadenovirus (Davison et al., 2003). Genome sizes within this family range from 26,163 bp (Atadenovirus) to 48,395 bp (Ichtadenovirus) (**Figure 1**). Aviadenovirus, specifically, is divided into 15 species based on factors such as phylogenetic distance, genetic and molecular analyses, host range, pathogenicity, and cross-neutralization.

Fowl adenovirus (FAdV) is particularly contagious in chickens, notably affecting broilers aged 3 to 5 weeks. Classified as a non-enveloped, double-stranded DNA virus, FAdV belongs to the Aviadenovirus genus within the Adenoviridae family. The genomes of different FAdV serotypes vary in size, ranging from approximately 43 to 45 kb, encoding approximately 10 structural and non-structural proteins. FAdVs are further categorized into five species (FAdV-A to FAdV-E) and 12 serotypes (FAdV-1–7, FAdV-8a/b, and FAdV-9–11) (**Table 1**). Since its initial outbreak in Pakistan in 1987, FAdV-4 has spread across Asia, Central and South America, and Europe (Grafl et al., 2012; Ravindran and Roy, 2014). The virus is responsible for hydropericardium syndrome (HPS), a severe disease in broiler chickens characterized by the accumulation of clear straw-colored fluid in the pericardial sac.

**Table 1:** List of FAdV species and serotypes.

Species	Serotype number			Proposed type strains	
	Europe	USA	ICTV	Europe	USA
A	1	1	1	CELO	QBV/Phelps
B	5	8	5	340	M2/Tipton
C	4	4	4	KR5	J2
C	11	10	10	C2B	C2B
D	2	2	2	GAL-1	P7
D	3	3	3	SR49	–
D	10	9	9	A2	A2
D	12	12	11	380	–
E	6	5	6	CR119	–
E	7	11	7	YR36/XII	XII
E	8	6	8a	TR59	T8
E	9	7	8b	764	B3

Inclusion body hepatitis (IBH), caused by FAdV serotypes -2 and -11 and -8a and -8b, presents with acute mortality and is often associated with anemia. Vertical and horizontal transmission routes contribute to the spread of IBH, with clinical signs including inappetence, comb pallor, depression, wattles, and ruffled feathers. Adenoviral gizzard erosion (AGE) is an emerging infection impacting broiler flock productivity (Tsiouris et al., 2022). FAdV-1 is a common causative agent for most AGE cases, inducing the condition in specific pathogen-free white leghorn chickens and industrial SPF broilers in experimental settings. The adenovirus capsid comprises three major structural proteins: hexon, fiber, and penton base. Hexon capsomers, penton bases, and fiber proteins, along with other structural proteins, contribute to the capsid’s structure (Sohaimi and Bejo, 2021). Hexon and fiber proteins, with variable lengths and exposed loops, form type-specific epitopes. Fiber proteins, including short and long fibers, vary in length and are attached to a single penton base (Chigbu and Labib, 2018). The penton base, essential for virion internalization, undergoes structural modifications during infection.

Many current approaches to vaccine development rely on either single antigens or diverse antigens administered in a single shot. However, epitope-based vaccines, which focus on the fundamental unit capable of triggering a cellular or humoral immune response within an antigen, are increasingly regarded as a promising futuristic strategy (Safavi et al., 2019; Kardani et al., 2020, 2021). A multi-epitopic vaccine consists of a sequence of epitopic (antigenic) peptides, aiding in infection prevention or the stimulation of an immune response. An ideal multi-epitopic vaccine is designed to include epitopes capable of eliciting immune responses from cytotoxic and helper T lymphocytes, as well as B cells against the targeted microorganism. Compared with traditional vaccines, multi-epitope-based vaccines offer advantages such as lower development costs, the absence of microbial culturing, and the ability to surpass numerous wet lab procedures, thereby saving time. Epitope-based vaccines also mitigate the risk associated with virulence reversal, a concern with live attenuated strains. Additionally, engineered and optimized epitopes in these vaccines enhance their effectiveness, and their small size contributes to increased chemical stability. These vaccines provide safety by not involving entire pathogens, offering specificity and stability. The presence of multiple epitopes allows the vaccine candidate to bind to multiple HLA alleles simultaneously, ensuring the desired immune response across a diverse population. Multi-epitopic vaccines have been created for various poultry pathogens, including Newcastle disease virus, avian influenza A (H7N9) virus (Hasan et al., 2019), Eimeria parasitic infection (Madlala et al., 2021), and *Mycoplasma gallisepticum* (Mugunthan and Mani Chandra, 2021; Mugunthan et al., 2024). The growing attention toward epitope-based vaccines is attributed to their specificity (Mugunthan and Harish, 2021).

Considering the crucial roles of hexon, fiber, and penton base proteins in FAdV, these proteins were selected for the construction of a multi-epitope vaccine candidate against FAdV infections in chickens.

The objectives of this study are multifaceted, aiming to address the pressing need for effective control measures against FAdV infections in chickens. Firstly, the study seeks to comprehensively understand the genomic diversity and classification of FAdV, including its various serotypes and their associated pathogenicity. Secondly, the study endeavors to develop a novel multi-epitope vaccine candidate targeting the crucial proteins, viz., hexon, penton base, and fiber, with the goal of inducing robust immune responses capable of preventing FAdV infections and associated diseases in poultry. Furthermore, the study aims to evaluate the feasibility and efficacy of epitope-based vaccine strategies compared with traditional approaches, considering factors such as cost-effectiveness, safety, and immunogenicity. Ultimately, the research endeavors to contribute to the advancement of poultry health management practices by providing a promising and innovative solution to combat FAdV infections in poultry.

## 2 Materials and methods

### 2.1 Sequence retrieval from NCBI

The complete genome sequence of fowl adenovirus was retrieved from the NCBI database (https://www.ncbi.nlm.nih.gov). The reference accession numbers of fowl adenovirus are FAdV A (NP_043882.1), FAdV B (YP_007985650.1), FAdV C (YP_004346925.1), FAdV D (NP_050284.1) and FAdV E (YP_004191817.1). From these reference accession numbers, the penton base, hexon, and fiber protein sequences were retrieved.

### 2.2 Identification of the conserved region and evolutionary analysis

The retrieved nucleotide sequence was compared to find the similarity using BLAST. Multiple sequence alignment was performed by clustal omega, and the phylogenetic relationship was analyzed with the help of phylogeny.fr.

### 2.3 Antigenicity and allergenicity and toxicity prediction

VaxiJen servers (http://www.ddg-pharmfac.net/vaxijen/) (Ong et al., 2020) and ANTIGENpro (http://scratch.proteomics.ics.uci.edu/) (Magnan et al., 2010) were used to detect the antigenicity of the protein sequence with a threshold of 0.5 for the virus. The allergenicity of the protein sequence was predicted by AllerTOP (https://www.ddg-pharmfac.net/AllerTOP/) (Dimitrov et al., 2013). The SOLpro was used for the calculation of the *Escherichia coli* host’s solubility (http://scratch.proteomics.ics.uci.edu/) (Cheng et al., 2005). The toxicity of the proteins was predicted by ToxinPred 2.0 (https://webs.iiitd.edu.in/raghava/toxinpred2/) (Gupta et al., 2013).

### 2.4 Analysis of physiochemical properties

Different physiochemical properties, including molecular weight (MW), isoelectric point (p*I*), stability index, half-life, aliphatic index, and grand average of hydropathicity (GRAVY), were estimated by using the ProtParam server (http://web.expasy.org/protparam) (Gasteiger et al., 2005).

### 2.5 Determination of the T-cell epitope

Both MHC-I and MHC-II T-cell epitopes were predicted by web-based servers. The prediction was based on high conservancy and good binding affinity (IC_50_; IC_50_ < 50 nM). The MHC-I epitope was predicted by the NetMHCcons tool (http://www.cbs.dtu.dk/services/) (Karosiene et al., 2012). The length was set as 9mer, and HLA-B*40:06, HLA-B*41:03, and HLA-B*41:04 alleles were used. Similarly, the NetMHCIIpan-1 tool (http://www.cbs.dtu.dk/services/NetMHCIIpan-3.1/) (Andreatta et al., 2015) was used to predict the MHC-II epitopes. The length was set as 15 mer peptide, and DRB1–1310, DRB1–1366, DRB1–1445, and DRB1–1482 alleles were selected for MHC-II epitope prediction.

### 2.6 Determination of the B-cell epitope

Identification and characterization of B-cell epitopes in target antigens is one of the key steps in epitope-driven vaccine design. The B-cell epitopes were predicted using the b-cell tool (http://tools.iedb.org/bcell/) (Larsen et al., 2006).

### 2.7 Vaccine construction

The multi-epitope vaccine ass constructed with an adjuvant avian β-defensin, MHC-I epitopes, MHC-II core epitopes, and B-cell epitopes that were linked using the linkers EAAAK, AAY, GPGPG, and KK.

### 2.8 Secondary and tertiary structure prediction and refinement

PSIPRED (http://bioinf.cs.ucl.ac.uk/psipred/) (Buchan and Jones, 2019) and GOR-4 (https://npsa-prabi.ibcp.fr/cgi-bin/npsa_automat.pl?page=npsa_gor4.html) (Garnier et al., 1996) were used to predict the secondary structure of the vaccine construct. I-TASSER (https://zhanglab.ccmb.med.umich.edu/I-TASSER/) (Yang et al., 2015) was used for predicting a three-dimensional structure model of the vaccine construct. Among the 10 templates, only the best template was chosen. The C-score, estimated TM score, and estimated RMSD were also calculated. The predicted tertiary structure was refined by the GalaxyRefine server (http://galaxy.seoklab.org/cgi-bin/submit.cgi?type=REFINE) (Heo et al., 2013) to improve the vaccine protein structure quality. GDT-HA, RMSD, MolProbity, Clash score, Poor rotamers, and Rama-favored scores were analyzed (Ko et al., 2012).

### 2.9 Structure validation

The refined tertiary structure was validated by the PROCHECK server (https://servicesn.mbi.ucla.edu/PROCHECK/) (Laskowski et al., 1993). PROCHECK analyzed the stereochemical quality of a protein structure, producing a number of PostScript plots analyzing its overall and residue-by-residue geometry. The Ramachandra plot is based on non-bonded interaction statistics between different atom types that are calculated by its refined structure database. It defines the stability of the constructed vaccine protein. ProSA was also used to validate the tertiary structure which checks 3D models of protein structures for potential errors (https://prosa.services.came.sbg.ac.at/prosa.php). The *Z*-score indicates overall model quality and measures the deviation of the total energy of the structure with respect to an energy distribution derived from random conformations. An NMR structure was calculated from the magnetic properties of several nuclei, while an X-ray structure was derived from the electron density of non-hydrogen atoms.

### 2.10 Immunological analyses for the chimeric multi-epitope vaccine

ElliPro (http://tools.iedb.org/ellipro/) (Ponomarenko et al., 2008) has been tested on a reference dataset of discontinuous epitopes inferred from 3D structures of antibody–protein complexes. Depending on the threshold values for parameters *R* of 6 Å and *S* of 0.5, the average number of predicted epitopes in each protein analyzed was 4, with a variance from 2 to 8. For continuous B-cell epitope prediction, BCEPred was used (https://webs.iiitd.edu.in/raghava/bcepred/index.Html) (Saha and Raghava, 2004), which also analyzes the four amino acid properties such as hydrophilicity, flexibility, polarity, and exposed surface. This server plots the residue properties along the protein backbone, assisting users in the rapid visualization of the B-cell epitope on the protein.

### 2.11 Molecular docking and dynamic simulation

The active sites were predicted using CPORT (De Vries and Bonvin, 2011). The molecular docking between the constructed vaccine candidate and the avian immune receptors and TLR5 was predicted by the HADDOCK server (https://wenmr.science.uu.nl/haddock2.4/) (Van Zundert et al., 2016). The best model was selected and refined for 100% water refinement, and the binding affinity of the protein–protein complex was predicted using the PRODIGY web server (Xue et al., 2016). The interacting residues between the docked complexes were mapped with the help of PDBsum (https://www.ebi.ac.uk/thorntonsrv/databases/pdbsum/Generate.html) (Laskowski et al., 2018). For the dynamic simulation, the iMODS server (https://imods.iqf.csic.es/) was used to perform the internal coordinates analysis based on the protein–protein structural complex. The server calculates a specific combined motion of a large macromolecule along with the NMA of dihedral coordinates.

### 2.12 *In silico* cloning

The constructed multi-epitope was codon-optimized to improve the bacterial expression of *Escherichia coli* K12 strain by using the web server JCAT (http://www.jcat.de/) (Grote et al., 2005). The optimized codons were inserted into the vector pET30a(+) using the SnapGene web tool (https://www.snapgene.com).

### 2.13 *In silico* immune simulation

The immune response generated by the constructed vaccine was performed by the web server C-ImmSim (http://kraken.iac.rm.cnr.it/C-IMMSIM/) (Castiglione et al., 2012). The parameters for the analyses were set to default. However, it offers the chance to test the overall immunogenicity of a generic protein sequence in the form of its amino acid sequence.

## 3 Results

### 3.1 Identification of the conserved region

Using the BLAST tool, the sequence of interest was compared with the available sequences in the database, revealing significant sequence similarities across various genomes. Further analysis with CLUSTAL O demonstrated that the retrieved protein sequences from the fowl adenovirus species exhibited a considerable number of conserved regions between the sequences (**Supplementary Figure 1**).

### 3.2 Estimation of physiochemical properties

The results obtained from the ProtParam server provided valuable insights into the physiochemical properties of the proteins analyzed. Firstly, the p*I* values, which range from 4.31 to 6.68, indicated that all proteins are acidic in nature. Next, the instability index was utilized to assess protein stability, with values above 40 indicating instability. It was observed that all penton base protein sequences exhibited instability, while hexon and fiber proteins were deemed stable. Additionally, the aliphatic index, ranging from 67.15 to 86.80, suggested that these proteins possess thermal stability. Moreover, the negative GRAVY values indicated that the proteins are predominantly non-polar. These findings are summarized in **Supplementary Table 1**, demonstrating the distinct physiochemical characteristics of the analyzed proteins.

### 3.3 Analyses of antigenicity, allergenicity, and toxicity

The antigenicity prediction using the VaxiJen server revealed that all species of fowl adenovirus analyzed met the threshold value of 0.4, indicating their antigenic nature. Furthermore, it was determined that all proteins were non-toxic, as shown in **Table 2**. In addition to antigenicity and toxicity, other properties such as protein localization, solubility, and the presence of signal peptides were predicted. From the results, it is evident that the penton base and hexon proteins were localized in the cytoplasm, while the fiber protein was found in the nucleus. Moreover, all proteins were predicted to be soluble and lacked signal peptides. These findings provided valuable insights into the characteristics and potential functions of the analyzed proteins in the context of fowl adenovirus infection.

**Table 2:** Antigenicity, allergenicity, localization, and toxicity prediction of protein sequences.

Protein	Species	VaxiJen score	Probability	Allergenicity	Localization	Signal peptide	Toxicity
Penton	A	0.4840	Antigenic	Non-allergen	Cytoplasm, soluble	No signal peptide	Non-toxin
	B	0.4706	Antigenic	Allergen	Cytoplasm, soluble	No signal peptide	Non-toxin
	C	0.5194	Antigenic	Allergen	Cytoplasm, soluble	No signal peptide	Non-toxin
	D	0.5130	Antigenic	Allergen	Cytoplasm, soluble	No signal peptide	Non-toxin
	E	0.4975	Antigenic	Non-allergen	Cytoplasm, soluble	No signal peptide	Non-toxin
Hexon	A	0.4241	Antigenic	Allergen	Cytoplasm, soluble	No signal peptide	Non-toxin
	B	0.4725	Antigenic	Allergen	Cytoplasm, soluble	No signal peptide	Non-toxin
	C	0.5070	Antigenic	Allergen	Cytoplasm, soluble	No signal peptide	Non-toxin
	D	0.4468	Antigenic	Non-allergen	Cytoplasm, soluble	No signal peptide	Non-toxin
	E	0.5083	Antigenic	Allergen	Cytoplasm, soluble	No signal peptide	Non-toxin
Fiber	A	0.5908	Antigen	Allergen	Nucleus, soluble	No signal peptide	Non-toxin
	B	0.6045	Antigen	Non-allergen	Cytoplasm, soluble	No signal peptide	Non-toxin
	C	0.6045	Antigen	Non-allergen	Cytoplasm, soluble	No signal peptide	Non-toxin
	D	0.5266	Antigen	Non-allergen	Nucleus, soluble	No signal peptide	Non-toxin
	E	0.6771	Antigen	Non-allergen	Nucleus, soluble	No signal peptide	Non-toxin

### 3.4 Allele identification

Due to the absence of chicken MHC alleles in the database, human MHC alleles were utilized as substitutes for both MHC-I and MHC-II in the immunoinformatics tool for predicting MHC-binding epitopes. This approach has been adopted in previous studies focusing on epitope prediction against poultry pathogens. It is worth noting that while chickens and humans possess different MHC alleles, there are similarities in their antigen presentation systems. Research has indicated that chicken B-F alleles exhibit the capability to induce immunological responses similar to human MHC class I alleles, particularly during antigen presentation (Ali et al., 2019; Aziz et al., 2019). This suggests that human alleles can serve as reasonable substitutes for predicting epitopes against poultry pathogens when chicken-specific alleles are unavailable.

### 3.5 Predictions of cytotoxic T lymphocytes

For the MHC-I epitope prediction, the penton base sequences were fragmented into possible 9mer peptide lengths using NetMHCcons. Likewise, for MHC-II prediction, NetMHCIIpan was employed. Subsequently, the peptides generated and the “best human substitute alleles” for MHC-I and MHC-II were utilized to predict the MHC–peptide binding affinity on their respective software platforms. From the results obtained, peptides from both MHC-I and MHC-II were identified where the MHC–peptide binding affinity (IC_50_; IC_50_ < 50 nM) was observed. These peptides are considered strong binding epitopes and are presented in **Supplementary Table 2** for MHC-I and in **Supplementary Table 3** for MHC-II. These epitopes hold significance in potential immunological responses and could be further investigated for their role in immune recognition and response against the fowl adenovirus.

### 3.6 Predictions of cytotoxic B lymphocytes

B-cell epitopes were predicted using BepiPred and ABCPred, with a window length of 16. Peptides exceeding the threshold value of 0.51 were considered B-cell binding epitopes, as shown in **Supplementary Table 4**. Among these predicted epitopes, the topmost epitope was identified as the B-cell epitope for potential vaccine construction. This epitope holds promise for eliciting an immune response against the fowl adenovirus and could serve as a key component in vaccine development efforts.

### 3.7 Construction of penton base epitope vaccine

A multi-epitopic vaccine against fowl adenovirus was constructed by combining predicted epitopes from MHC-I, MHC-II, and B cells. These epitopes were linked together using various linkers including EAAAK, KK, and AAY. Additionally, avian β-defensin was incorporated as an adjuvant to enhance the immunogenicity capacity of the vaccine. Consequently, the FAdV epitope vaccine comprised 493 amino acids. Following the construction of the vaccine, further validation was conducted to assess its physicochemical properties, allergenicity, and antigenicity. This validation process ensures the safety, efficacy, and potential effectiveness of the vaccine candidate against fowl adenovirus infection.

### 3.8 Analyses of physiochemical properties, antigenicity, and allergenicity

The molecular characteristics and properties of the penton base epitope vaccine were analyzed to assess its suitability and efficacy. The molecular weight of the vaccine was determined to be 53.4 kDa, indicating its relatively large size. Furthermore, the p*I* was calculated to be 9.22, suggesting that the vaccine was alkaline in nature. Moreover, the vaccine exhibited favorable characteristics regarding stability, thermostability, and hydrophilicity. The instability index was estimated at 22.91, indicating a stable protein structure. The aliphatic index, calculated to be 81.83, suggested that the vaccine was highly thermostable. Additionally, the grand average of hydropathicity was determined to be −0.286, indicating a hydrophilic nature.

The predicted probability of antigenicity using the ANTIGENpro server was 0.704966, suggesting a high likelihood of inducing an immune response. Similarly, the SOLpro server predicted the vaccine to be soluble with a probability of 0.590493, further supporting its potential as a viable vaccine candidate. Furthermore, allergenicity prediction using AllerTOP indicated that the penton base epitope vaccine was non-allergenic, emphasizing its safety profile for potential use in vaccination strategies against fowl adenovirus.

### 3.9 Secondary and tertiary structure prediction

The secondary structure of the vaccine candidate was predicted using PSIPRED, as illustrated in **Supplementary Figure 2**. The analysis revealed that the protein predominantly consisted of a random coil (41.38%), followed by alpha helices (45.84%) and extended strands (12.78%).

**Figure 2:**
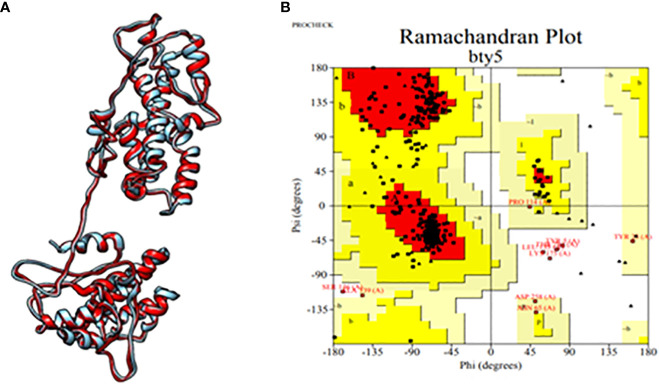
**(A)** Tertiary structure of the constructed multi-epitopic vaccine and **(B)** Ramachandran plot for validation.

Subsequently, the tertiary structure of the designed epitopic vaccine was predicted using I-TASSER. Among the top 10 templates provided by I-TASSER, the first model was selected based on a C-score of 1.86, with an estimated TM score of 0.49 ± 0.15 and an estimated RMSD value of 11.4 ± 4.5 Å. However, the selected model exhibited a Rama-favored region of only 68.39%. To improve the model, refinement was performed using GalaxyRefine, resulting in the generation of the top 5 models. Among these, model 3 was identified as the best model, possessing the highest Rama-favored region along with other favorable parameters including GDA-HT: 0.9035, RMSD: 0.543, MolProbity: 2.142, Clash score: 13.2, and Poor rotamers: 0.3. Furthermore, analysis using PROCHECK revealed that 91.2% of the residues were located within the most favored regions, as depicted in **Figure 2**. These results indicate the successful prediction and refinement of the tertiary structure of the epitopic vaccine, providing valuable insights into its structural characteristics and stability.

### 3.10 Immunological property assessment

The BCEPred server was employed to predict the continuous epitopes of B cells in the multi-epitope vaccine, using default parameters. Additionally, vital properties such as accessibility, polarity, flexibility, hydrophilicity, antigenicity, and exposed surfaces were predicted for the epitopes, as outlined in **Table 3**.

**Table 3:** List of predicted B-cell epitopes.

S. no.	Parameters	Epitopes
1	Hydrophilicity	28–31, 33, 69–70, 243–244, 253–255, 276–277, 313, 318–321, 391, 411–412, 436, 440, 454–456, 473–491
2	Flexibility	241–242, 251–253, 274–275, 277, 286, 308, 318–320, 360, 362–363, 374, 454, 458–459, 461, 470, 474–489
3	Accessibility	25–30, 65, 68–70, 109–110, 179, 189, 191–193, 218–223, 233–234, 238–239,243–250, 252–256, 258, 266, 271–272, 276–283, 288–294, 299–304, 310–327, 332–333, 337–338, 342–343, 348, 354–355, 357–366, 375–378, 400, 473
4	Turns	42, 426
5	Surface	221–223, 243–245, 254–256, 266, 276–282, 291–293, 300–304, 311–316, 320–322, 324, 326, 332, 337–338, 343, 347–348, 357–360, 365–366, 376
6	Polar	42–48, 50, 70, 73–75, 179–182, 195, 233–234, 243–245, 248, 254–256, 266–267, 276–281, 283, 288–292, 301–304, 312–316, 320–334, 347–348, 359–360, 365–366, 376–378
7	AntiPro	4–5, 9, 13–17, 40–41, 42–46, 56–63, 112, 169–172, 196, 224, 235–237, 259–263, 344–346, 358, 405–408, 447

**Figure 3:**
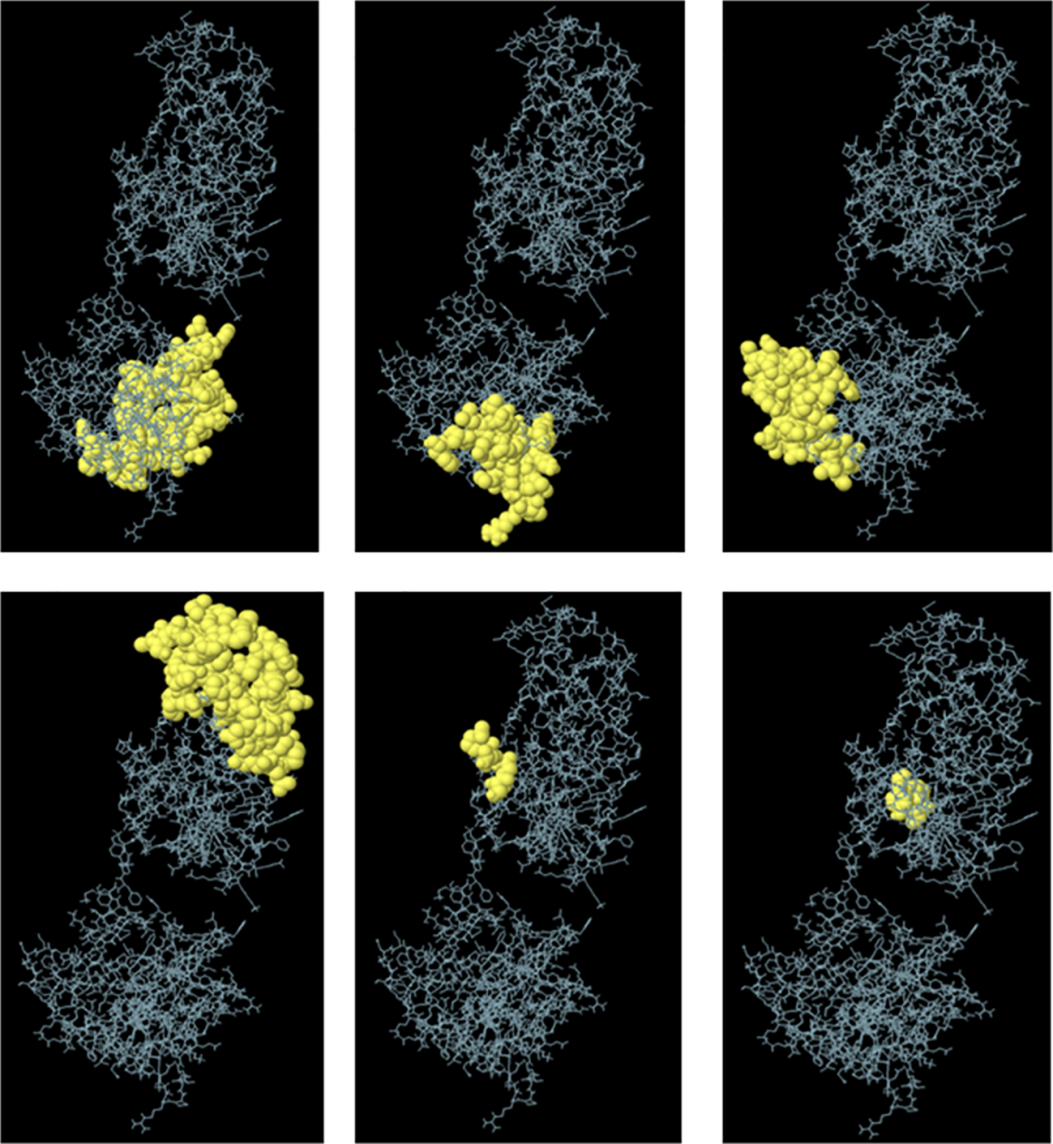
Predicted conformational B-cell epitopes by the ElliPro tool. The immunogenic epitopes are depicted as yellow globules on the ball and stick representation of multi-epitopic vaccine construct structure.

Furthermore, ElliPro was utilized for the discontinuous prediction of B-cell epitopes, with parameters set to a minimum score of 0.5 and a maximum distance of 6 Å. This analysis resulted in the prediction of six discontinuous epitopes, which are detailed in **Supplementary Table 5** and visualized in **Figure 3**. These predicted epitopes provide valuable information regarding potential regions of the vaccine candidate that could induce B-cell immune responses, thereby aiding in the design and optimization of the vaccine for enhanced efficacy against fowl adenovirus.

### 3.11 Molecular docking and dynamic simulation

**Figure 4:**
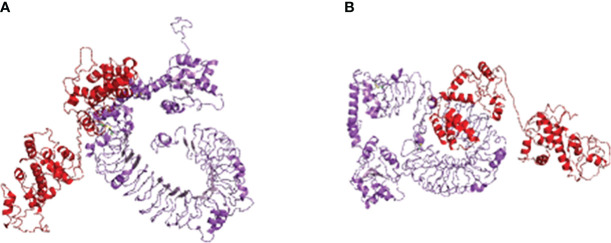
Molecular docking of multi-epitopic vaccines with chicken immune receptor TLR2 **(A)** and TLR5 **(B)**.

Using HADDOCK, the multi-epitope vaccine was docked with avian immune receptors TLR2 and TLR5. For TLR2, HADDOCK clustered 149 structures into 17 clusters, with 37.27% of water-refined models, as depicted in **Figure 4A** and detailed in **Table 4**. Similarly, for TLR5, HADDOCK clustered 156 structures into five clusters, with water-refined models accounting for 39.47%, as illustrated in **Figure 4B** and outlined in **Table 5**.

**Table 4:** Hydrogen bond interaction between TLR2 and multi-epitopic vaccine.

TLR2 receptor		Multi-epitopic vaccine	
Residue no.	Residue name	Residue no.	Residue name
467	LYS	213	ASP
492	GLU	215	LYS
516	TYR	213	ASP
516	TYR	214	ASN
522	GLN	8	ALA
601	ASN	69	ALA
629	TYR	62	LEU
673	TRP	89	SER
674	TYR	66	MET
675	LYS	73	TYR

**Table 5:** Hydrogen bond interaction between TLR5 and multi-epitopic vaccine.

TLR5 receptor		Multi-epitopic vaccine	
Residue no.	Residue name	Residue no.	Residue name
143	GLN	63	PHE
192	ASP	65	ASN
219	ARG	58	TYR
245	ASP	234	TYR
377	HIS	73	TYR
404	SER	77	VAL
405	LYS	121	TYR
405	LYS	48	GLU

From both sets of results, the model with the highest negative score was selected and refined using the HADDOCK refinement server, resulting in HADDOCK clustering 20 structures into one cluster for both TLR2 and TLR5. The HADDOCK scores for TLR2 and TLR5 were recorded as −397.3 ± 3.7 and −358.7 ± 9.3, respectively. These higher negative values indicate a strong binding affinity between the receptors and the vaccine.

Furthermore, large-scale mobility and stability of macromolecules were investigated through normal mode analysis (NMA) via the iMODS server. The server calculates a specific combined motion of a large macromolecule along with NMA of dihedral coordinates of Cα atoms. The eigenvalues for the TLR2 docked complex (**Supplementary Figure 3**) and TLR5 docked complex (**Supplementary Figure 4**) were 1.713223e−06 and 2.784556e−06, respectively. These eigenvalues provide insights into the flexibility and stability of the docked complexes, aiding in understanding their potential interactions and functionality.

### 3.12 *In silico* cloning

The multi-epitopic protein sequence was reverse-transcribed into cDNA, resulting in a sequence size of 1,479 base pairs (bp). Subsequently, the sequence was codon-optimized for bacterial expression in *E. coli* using the JCAT tool. The Codon Adaptation Index (CAI) value of the optimized sequence was determined to be 0.96, indicating high translational efficiency. Additionally, the GC content of the optimized sequence in *E. coli* was calculated to be 50.30%.

**Figure 5:**
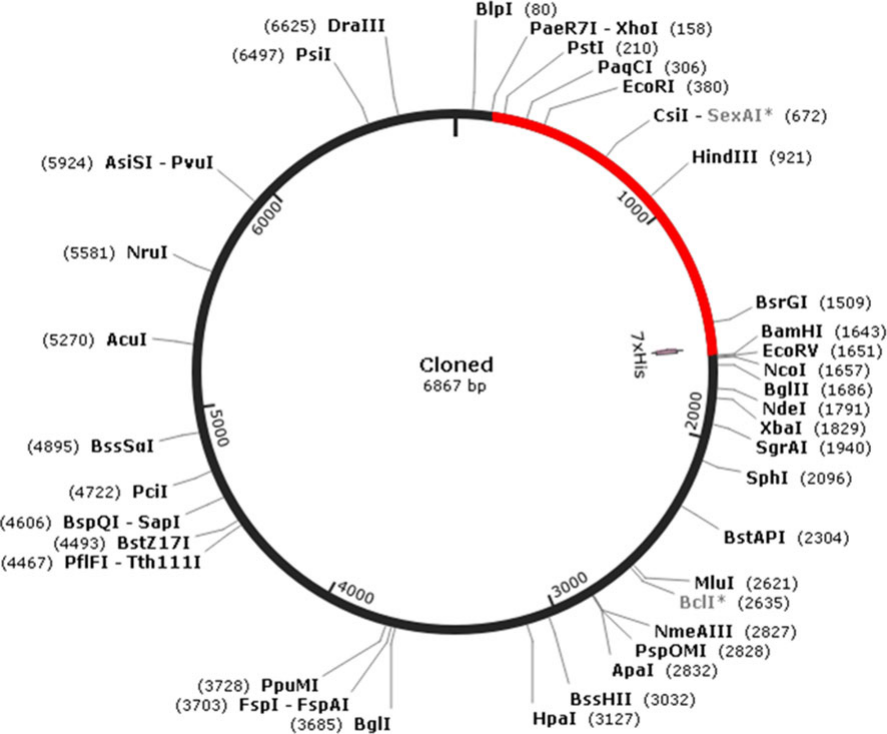
*In silico* cloning of multi-epitopic vaccine.

To facilitate *in silico* cloning, the presence of restriction enzyme sites within the penton base epitope vaccine constructed sequence was examined. It was found that the restriction enzyme sites *Xho*I and *Bam*HI were absent in the vaccine sequence. Therefore, these specific restriction enzyme sites were selected for cloning the multi-epitopic sequence into the pET30a(+) vector, which has a size of 5,388 bp (**Figure 5**). As a result of the cloning process, the final clone size was determined to be 6,867 bp, incorporating the multi-epitopic sequence into the vector for expression in bacterial systems. This cloning strategy ensures efficient expression and production of the multi-epitope vaccine candidate in *E. coli.*

### 3.13 *In silico* immune simulation

**Figure 6:**
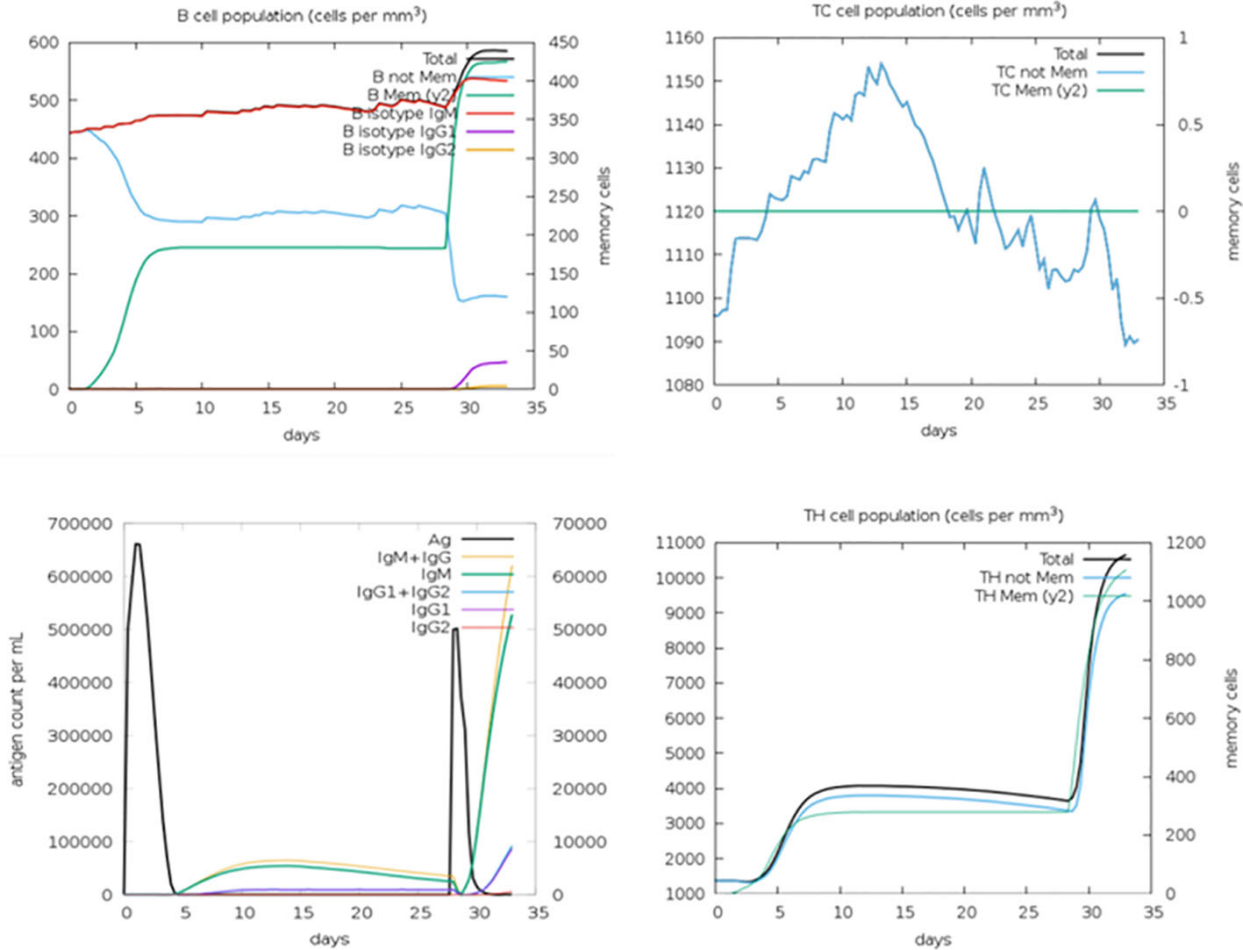
*In silico* immune simulation showing B-cell population, TC cell population, antigen count, and TH cell population.

The *in silico* immune simulation conducted aimed to analyze the host’s immune response upon interaction with the multi-epitope vaccine. Through this simulation, the characteristics of B cells and T cells in the host were elucidated. The results of the immune simulation revealed that the host’s immune response was activated following the administration of the multi-epitope vaccine. Subsequently, both B-cell and T-cell counts were observed to increase significantly and remained active for an extended period, exceeding 50 days. Additionally, the simulation demonstrated that memory B cells and memory T cells remained constant throughout the observation period (**Figure 6**).

These findings indicate that the multi-epitope vaccine effectively triggers the immune response in the host, leading to the activation and proliferation of both B cells and T cells. Furthermore, the maintenance of memory B cells and memory T cells suggests the establishment of long-lasting immune memory, which could confer protection against future encounters with the fowl adenovirus. Overall, the *in silico* immune simulation provides valuable insights into the potential efficacy of the multi-epitope vaccine in inducing a robust and sustained immune response in the host.

## 4 Discussion

Fowl adenovirus, responsible for inducing hydropericardium, inclusion bodies, and gizzard erosion in *Gallus gallus domesticus*, poses challenges in traditional vaccine development due to its time-consuming, costly, and potentially unreliable nature. To address these issues, this study employed immunoinformatics methods to design a vaccine that is cost-effective, rapid, and highly accurate, fostering an enhanced immune response against the targeted disease. The present study aimed to design and evaluate a multi-epitope vaccine candidate against fowl adenovirus using a comprehensive *in silico* approach. Through a series of computational analyses, we assessed various aspects of the vaccine candidate, including epitope prediction, physicochemical properties, docking with immune receptors, and *in silico* immune simulation.

Vaccines play a crucial role in providing host organisms with protection against specific diseases. However, the conventional process of vaccine development is highly meticulous, expensive, and time-consuming. Immunoinformatics tools offer a solution by enabling the rapid design and development of vaccines with enhanced specificity and ease. These tools facilitate the identification of pathogens and their associated immunogenic proteins, as well as the prediction of diverse immune-dominant epitopes crucial for eliciting humoral and cell-mediated immune responses against the pathogen. Consequently, multi-epitope-based peptide vaccines can be designed utilizing immunogenic proteins from the pathogen.

Currently, a large number of peptide vaccines are under development, with the majority targeting human infectious diseases and tumors (Kolesanova et al., 2019; Kar et al., 2020). However, there is a notable lack of research in the field of *in silico* vaccines for poultry and animals. Previous studies have focused on predicting epitopes for animal diseases such as foot and mouth disease (Liu et al., 2017) and animal trypanosomiasis (Michel-Todó et al., 2020), demonstrating the effectiveness of multi-epitopic vaccines compared with currently available options.

Further validation of the immunoinformatics approach has been conducted to design multi-epitopic vaccines against infectious diseases in poultry (Osman et al., 2016; Hasan et al., 2019). This validation process includes evaluating complete antigenic epitopes and utilizing molecular modeling to assess potential binding with host proteins. *In silico* validations, such as molecular docking, *in silico* cloning, and immune simulation, have been integrated into these studies to ensure that the constructed multi-epitope vaccines function effectively as candidates against infections such as FAdV.

The primary objective of multi-epitopic vaccines is to identify specific CTL, HTL, and B-cell epitopes within antigenic proteins that induce targeted and effective immune responses. Initial analyses of antigenicity and allergenicity confirm that selected proteins are antigenic and non-allergenic. Additionally, the predicted epitopes were evaluated for allergenicity, solubility, and other physicochemical properties, ensuring their safety and suitability for vaccine development. Moreover, vaccine design necessitates the inclusion of an adjuvant to enhance vaccine efficacy.

The multi-epitope vaccine constructed from these epitopes was designed to enhance immunogenicity by incorporating adjuvants and linkers. Avian β-defensin was included as an adjuvant to improve the vaccine’s efficacy, while linkers such as EAAAK, KK, and AAY were used to connect the epitopes. The resulting vaccine exhibited favorable physicochemical properties, including stability, solubility, and non-allergenicity, indicating its potential as a safe and effective vaccine candidate. Docking studies were conducted to assess the interaction between the multi-epitope vaccine and avian immune receptors TLR2 and TLR5. HADDOCK analysis revealed strong binding affinity between the vaccine and both receptors, suggesting the potential of the vaccine to stimulate the innate immune response upon administration.

Furthermore, *in silico* immune simulation was performed to evaluate the host’s immune response to the multi-epitope vaccine. The simulation demonstrated the activation of B cells and T cells in response to the vaccine, with both cell types exhibiting increased counts and sustained activity for more than 50 days. Additionally, memory B cells and memory T cells remained constant, indicating the establishment of long-term immune memory.

One significant challenge arises from epitope prediction tools, which often fail to adequately account for the necessity of identifying precise antigen processing sites. These sites are crucial for the accurate prediction and presentation of epitopes. Given that antigen processing mechanisms can differ based on proinflammatory signals and among different cell types, existing prediction algorithms may not effectively assess the processing efficiency of viral antigens within specific infected cells (Silva-Arrieta et al., 2020).

Overall, the *in silico* analyses presented in this study provide promising evidence for the effectiveness of the multi-epitope vaccine against fowl adenovirus. However, further experimental validation, including *in-vitro* and *in-vivo* studies, is necessary to confirm the vaccine’s efficacy and safety before clinical application. Additionally, future studies may focus on optimizing the vaccine design and formulation to enhance its immunogenicity and protective efficacy against fowl adenovirus infection.

## 5 Conclusion

Due to the increase of infection and the absence of efficient vaccines, there have recently been various attempts to develop vaccines against FAdV. This study predicted the potential CTL, HTL, and B-cell epitopes from the structural proteins of fowl adenovirus and designed a multi-epitope vaccine candidate. This approach of vaccine development will have advantages over the classical vaccine development methods by using small epitopes that will bind specifically to the paratopic region of the host immune cell and elicit a higher immune response. The results reported in this study could be supported by *in-vivo* and *in-vitro* laboratory evaluations in the future. Nevertheless, these findings provide a novel way to develop a vaccine against all the serotypes of FAdV.

## Data availability statement

The original contributions presented in the study are included in the article/**Supplementary Material**, further inquiries can be directed to the corresponding author/s.

## Author contributions

SM: Conceptualization, Formal analysis, Investigation, Methodology, Writing – original draft. DV: Conceptualization, Formal analysis, Investigation, Methodology, Writing – original draft. CG: Supervision, Validation, Writing – review & editing. DS: Validation, Writing – review & editing, Software. MH: Conceptualization, Data curation, Supervision, Validation, Writing – review & editing.
