# Supplementary material for: Systems approach to design multi-epitopic peptide vaccine candidate against fowl adenovirus structural proteins for Gallus gallus domesticus
Source: Front Cell Infect Microbiol. 2024 May 28;14:1351303. doi: 10.3389/fcimb.2024.1351303 (PMC11177691; doi:10.3389/fcimb.2024.1351303)
Supplement: Supplementary file 1 [file DataSheet_1.docx]

Supplementary Table 1: Physiochemical properties like Number of amino acids, Molecular weight, Isoelectric point, Extinction Coeffcient, Half Life (Hrs), Instability Index, Aliphatic index and GRAVY

| **PROTEINS** | **SPECIES** | **AMINO ACIDS** | **MOL.WT** | **PI** | **EXTINCTION COEFFICIENT** | **HALF LIFE** | | | **INSTABILITY** | **ALIPHATIC INDEX** | **GRAVY** |
| --- | --- | --- | --- | --- | --- | --- | --- | --- | --- | --- | --- |
|  |  |  |  |  |  | **MAMMALS** | **YEAST** | **E. COLI** |  |  |  |
| PENTON | A | 515 | 56722.11 | 5.44 | 54125 | 30 | >20 | >10 | 49.52 | 81.94 | -0.342 |
|  | B | 542 | 59422.01 | 5.23 | 63970 | 30 | >20 | >10 | 47.79 | 83.63 | -0.269 |
|  | C | 525 | 57405.68 | 5.03 | 71210 | 30 | >20 | >10 | 46.3 | 79.28 | -0.3 |
|  | D | 570 | 62421.54 | 6.68 | 58595 | 30 | >20 | >10 | 49.54 | 80.05 | -0.316 |
|  | E | 552 | 59829.45 | 5.37 | 55615 | 30 | >20 | >10 | 48.46 | 82.5 | -0.244 |
| HEXON | A | 942 | 106709.25 | 5.31 | 181660 | 30 | >20 | >10 | 33.76 | 70.24 | -0.44 |
|  | B | 953 | 107518.2 | 5.16 | 182120 | 30 | >20 | >10 | 33.53 | 67.47 | -0.432 |
|  | C | 937 | 106008.37 | 5.19 | 187620 | 30 | >20 | >10 | 31.22 | 67.15 | -0.436 |
|  | D | 542 | 60319.91 | 6.58 | 66240 | 30 | >20 | >10 | 46.46 | 80.17 | -0.431 |
|  | E | 948 | 107276.78 | 5.13 | 179600 | 30 | >20 | >10 | 34.1 | 68.51 | -0.456 |
| FIBER | A | 410 | 42941.51 | 4.95 | 29005 | 30 | >20 | >10 | 27.09 | 86.8 | 0.037 |
|  | B | 554 | 58829.75 | 4.33 | 63050 | 30 | >20 | >10 | 26.81 | 84.12 | -0.143 |
|  | C | 554 | 58829.75 | 4.33 | 63050 | 30 | >20 | >10 | 26.81 | 84.12 | -0.143 |
|  | D | 571 | 60240.66 | 4.42 | 60195 | 30 | >20 | >10 | 29.2 | 77.18 | -0.245 |
|  | E | 523 | 55130.3 | 4.31 | 55600 | 30 | >20 | >10 | 25.3 | 80.76 | -0.185 |

Supplementary Table 2: List of predicted MHCI binding epitopes of different alleles and their affinity and percentage rank.

| **Protein** | **Species** | **Position** | **Allele** | **Peptide** | **Identity 1-log50k(aff)** | **Affinity(nM)** | **%Rank** |
| --- | --- | --- | --- | --- | --- | --- | --- |
| PENTON | B | 5 | HLA-B40:06 | NEFGHLEAV | 0.510 | 201.02 | 0.15 |
|  |  |  | HLA-B41:03 |  | 0.594 | 80.87 | 0.50 |
|  |  |  | HLA-B41:04 |  | 0.514 | 192.00 | 0.50 |
|  | C | 324 | HLA-B40:06 | IELDNAAPL | 0.416 | 556.30 | 0.50 |
|  |  |  | HLA-B41:03 |  | 0.694 | 27.41 | 0.05 |
|  |  |  | HLA-B41:04 |  | 0.593 | 81.80 | 0.15 |
|  | D | 370 | HLA-B41:03 | IELADAKPL | 0.633 | 52.74 | 0.25 |
|  |  |  | HLA-B41:04 |  | 0.512 | 196.55 | 0.50 |
|  | E | 352 | HLA-B41:03 | IELADAKPL | 0.693 | 27.71 | 0.05 |
|  |  |  | HLA-B41:04 |  | 0.558 | 118.99 | 0.25 |
| HEXON | A | 329 | HLA-B40:06 | TELSYQYML | 0.455 | 364.78 | 0.30 |
|  |  |  | HLA-B41:03 |  | 0.681 | 31.55 | 0.07 |
|  |  |  | HLA-B41:04 |  | 0.618 | 62.47 | 0.10 |
|  | B | 131 | HLA-B40:06 | REALFNNML | 0.449 | 389.10 | 0.30 |
|  |  |  | HLA-B41:03 |  | 0.673 | 34.59 | 0.10 |
|  |  |  | HLA-B41:04 |  | 0.647 | 45.61 | 0.08 |
|  |  | 190 | HLA-B40:06 | SEMPALATA | 0.536 | 151.51 | 0.10 |
|  |  |  | HLA-B41:03 |  | 0.611 | 67.28 | 0.40 |
|  |  |  | HLA-B41:04 |  | 0.598 | 77.70 | 0.15 |
|  |  | 721 | HLA-B40:06 | FEIKRNISI | 0.511 | 198.89 | 0.15 |
|  |  |  | HLA-B41:03 |  | 0.633 | 53.32 | 0.25 |
|  |  |  | HLA-B41:04 |  | 0.583 | 91.10 | 0.17 |
|  | C | 241 | HLA-B40:06 | TEYLGAVAV | 0.524 | 172.04 | 0.40 |
|  |  |  | HLA-B41:03 |  | 0.643 | 47.59 | 0.20 |
|  |  |  | HLA-B41:04 |  | 0.524 | 172.04 | 0.40 |
|  |  | 325 | HLA-B40:06 | TELSYQYML | 0.455 | 364.78 | 0.30 |
|  |  |  | HLA-B41:03 |  | 0.681 | 31.55 | 0.07 |
|  |  |  | HLA-B41:04 |  | 0.618 | 62.47 | 0.10 |
|  |  | 707 | HLA-B40:06 | FEIKRSVAL | 0.480 | 277.09 | 0.20 |
|  |  |  | HLA-B41:03 |  | 0.727 | 19.18 | 0.01 |
|  |  |  | HLA-B41:04 |  | 0.687 | 29.61 | 0.05 |
|  | D | 76 | HLA-B40:06 | REIANASLL | 0.430 | 474.36 | 0.40 |
|  |  |  | HLA-B41:03 |  | 0.677 | 32.94 | 0.07 |
|  |  |  | HLA-B41:04 |  | 0.608 | 69.13 | 0.12 |
|  |  | 261 | HLA-B40:06 | QEMLFRYLM | 0.498 | 227.76 | 0.40 |
|  |  |  | HLA-B41:03 |  | 0.665 | 37.71 | 0.12 |
|  |  |  | HLA-B41:04 |  | 0.639 | 49.90 | 0.08 |
|  | E | 131 | HLA-B40:06 | REAFFNNWI | 0.470 | 309.15 | 0.25 |
|  |  |  | HLA-B41:03 |  | 0.612 | 66.56 | 0.40 |
|  |  |  | HLA-B41:04 |  | 0.566 | 109.06 | 0.25 |
|  |  |  | HLA-B40:06 | TELSYQYML | 0.455 | 364.78 | 0.30 |
|  |  |  | HLA-B41:03 |  | 0.681 | 31.55 | 0.07 |
|  |  |  | HLA-B41:04 |  | 0.618 | 62.47 | 0.10 |
|  |  | 716 | HLA-B40:06 | FEIKRHVGI | 0.453 | 370.22 | 0.30 |
|  |  |  | HLA-B41:03 |  | 0.605 | 71.41 | 0.40 |
|  |  |  | HLA-B41:04 |  | 0.516 | 187.79 | 0.50 |
| FIBRE | A | 25 | HLA-B40:06 | GELDLVYPF | 0.513 | 194.51 | 0.15 |
|  |  |  | HLA-B 41:03 |  | 0.708 | 23.56 | 0.03 |
|  |  |  | HLA-B41:04 |  | 0.627 | 56.49 | 0.10 |
|  |  | 270 | HLA-B40:06 | NENARYFTV | 0.400 | 658.26 | 0.50 |
|  |  |  | HLA-B 41:03 |  | 0.597 | 77.86 | 0.50 |
|  |  |  | HLA-B41:04 |  | 0.500 | 224.54 | 0.50 |
|  | B | 193 | HLA-B40:06 | HEYELGLKL | 0.526 | 169.61 | 0.12 |
|  |  |  | HLA-B 41:03 |  | 0.657 | 40.68 | 0.15 |
|  |  |  | HLA-B41:04 |  | 0.610 | 68.03 | 0.12 |
|  | C | 193 | HLA-B40:06 | HEYELGLKL | 0.536 | 152.13 | 0.10 |
|  |  |  | HLA-B 41:03 |  | 0.668 | 36.31 | 0.12 |
|  |  |  | HLA-B41:04 |  | 0.568 | 106.70 | 0.25 |
|  |  | 231 | HLA-B40:06 | QEYELGVHL | 0.504 | 214.72 | 0.15 |
|  |  |  | HLA-B 41:03 |  | 0.659 | 40.24 | 0.15 |
|  |  |  | HLA-B41:04 |  | 0.563 | 113.68 | 0.25 |
|  |  | 473 | HLA-B40:06 | GEILTFNPV | 0.598 | 77.35 | 0.03 |
|  |  |  | HLA-B 41:03 |  | 0.639 | 49.70 | 0.25 |
|  |  |  | HLA-B41:04 |  | 0.652 | 43.12 | 0.08 |
|  | D | 215 | HLA-B40:06 | LEIDSQTMV | 0.433 | 462.65 | 0.40 |
|  |  | 378 | HLA-B 41:03 | AENSSVFKF | 0.602 | 74.16 | 0.50 |
|  |  | 423 |  | AENARYFTF | 0.675 | 33.66 | 0.10 |
|  |  | 423 | HLA-B41:04 | AENARYFTF | 0.664 | 38.01 | 0.05 |
|  | E | 47 | HLA-B41:03 | DELDLVYPF | 0.591 | 83.54 | 0.50 |
|  |  | 119 |  | LELTNQQQL | 0.627 | 56.59 | 0.30 |
|  |  | 119 | HLA-B41:04 |  | 0.507 | 208.39 | 0.50 |
|  |  | 375 |  | YQNARYFTF | 117.01 | 117.01 | 0.25 |

Supplementary Table 3: List of predicted MHC II binding epitopes along with core protein

| **Protein** | **Species** | **Position** | **Peptide** | **Core protein** |
| --- | --- | --- | --- | --- |
| **PENTON** | **A** | **70** | **CQNTTKFFYVDNKLSD** | **FYVDNKLSD** |
|  |  | **71** | **QNTTKFFYVDNKLSDL** |  |
|  |  | **72** | **NTTKFFYVDNKLSDLD** |  |
|  |  | **73** | **TTKFFYVDNKLSDLDT** |  |
|  |  | **74** | **TKFFYVDNKLSDLDTY** |  |
|  |  | **75** | **KFFYVDNKLSDLDTYN** |  |
|  |  | **218** | **SDIGVKFDTRYLDLLK** | **VKFDTRYLD** |
|  |  | **219** | **DIGVKFDTRYLDLLKD** |  |
|  |  | **269** | **LSLLLGIAKREPYSKG** | **LGIAKREPY** |
|  |  | **311** | **QDEDVIVVADARPLLK** | **IVVADARPL** |
|  |  |  |  | **VVADARPLL** |
|  |  | **312** | **DEDVIVVADARPLLKD** |  |
|  |  | **313** | **EDVIVVADARPLLKDS** |  |
|  |  | **314** | **DVIVVADARPLLKDSK** |  |
|  |  | **474** | **LKNSLSGLQRVLITDD** | **LSGLQRVLI** |
|  |  | **475** | **KNSLSGLQRVLITDDR** |  |
|  | **B** | **67** | **ADSELFVPVQRVMAPT** | **FVPVQRVMA** |
|  |  | **68** | **DSELFVPVQRVMAPTG** |  |
|  |  | **69** | **SELFVPVQRVMAPTGG** |  |
|  |  | **96** | **NRNTTKLFYVDNKLSD** | **FYVDNKLSD** |
|  |  | **97** | **RNTTKLFYVDNKLSDI** |  |
|  |  | **98** | **NTTKLFYVDNKLSDIE** |  |
|  |  | **99** | **TTKLFYVDNKLSDIET** |  |
|  |  | **100** | **TKLFYVDNKLSDIETY** |  |
|  |  | **101** | **KLFYVDNKLSDIETYN** |  |
|  |  | **295** | **RLSLMLGIAKRQPYAK** | **LGIAKRQPY** |
|  |  | **296** | **LSLMLGIAKRQPYAKG** |  |
|  |  | **297** | **SLMLGIAKRQPYAKGF** |  |
|  |  | **339** | **DEDVIVLADARPLLKD** | **IVLADARPL** |
|  |  | **340** | **EDVIVLADARPLLKDA** | **VLADARPLL** |
|  |  | **341** | **DVIVLADARPLLKDAK** |  |
|  |  | **500** | **PLKNSLAGLQRVLITD** | **LAGLQRVLI** |
|  |  | **501** | **LKNSLAGLQRVLITDD** |  |
|  |  | **502** | **KNSLAGLQRVLITDDQ** |  |
|  | **C** | **75** | **CRNTTKLFYVDNKASD** | **FYVDNKASD** |
|  |  | **76** | **RNTTKLFYVDNKASDI** |  |
|  |  | **77** | **NTTKLFYVDNKASDID** |  |
|  |  | **78** | **TTKLFYVDNKASDIDT** |  |
|  |  | **79** | **TKLFYVDNKASDIDTY** |  |
|  |  | **80** | **KLFYVDNKASDIDTYN** |  |
|  | **D** | **13** | **RGVTRELTAISRAMLG** | **LTAISRAML** |
|  |  | **14** | **GVTRELTAISRAMLGH** |  |
|  |  | **15** | **VTRELTAISRAMLGHH** |  |
|  |  | **16** | **TRELTAISRAMLGHHD** |  |
|  |  | **92** | **DTSELFVPVQRVMAPT** | **FVPVQRVMA** |
|  |  | **93** | **TSELFVPVQRVMAPTG** |  |
|  |  | **94** | **SELFVPVQRVMAPTGG** |  |
|  |  | **121** | **CQNTTKLFYVDNKLSD** | **FYVDNKLSD** |
|  |  | **122** | **QNTTKLFYVDNKLSDI** |  |
|  |  | **123** | **NTTKLFYVDNKLSDID** |  |
|  |  | **124** | **TTKLFYVDNKLSDIDT** |  |
|  |  | **125** | **TKLFYVDNKLSDIDTF** |  |
|  |  | **126** | **KLFYVDNKLSDIDTFN** |  |
|  |  | **528** | **PLKNSLAGLQRVLITD** | **LAGLQRVLI** |
|  |  | **529** | **LKNSLAGLQRVLITDD** |  |
|  |  | **530** | **KNSLAGLQRVLITDDQ** |  |
|  | **E** | **73** | **NGTELFVPVQRVMAPT** | **FVPVQRVMA** |
|  |  | **74** | **GTELFVPVQRVMAPTG** |  |
|  |  | **75** | **TELFVPVQRVMAPTGG** |  |
|  |  | **102** | **CQNTTKLFYVDNKLSD** | **FYVDNKLSD** |
|  |  | **103** | **QNTTKLFYVDNKLSDI** |  |
|  |  | **104** | **NTTKLFYVDNKLSDID** |  |
|  |  | **105** | **TTKLFYVDNKLSDIDT** |  |
|  |  | **106** | **TKLFYVDNKLSDIDTF** |  |
|  |  | **107** | **KLFYVDNKLSDIDTFN** |  |
|  |  | **510** | **PLKNSLAGLQRVLITD** | **LAGLQRVLI** |
|  |  | **511** | **LKNSLAGLQRVLITDD** |  |
|  |  | **512** | **KNSLAGLQRVLITDDQ** |  |
| **HEXON** | **A** | **447** | **YLPDKYKFSIRGFDP** | **YKFSIRGFD** |
|  |  | **448** | **LPDKYKFSIRGFDPV** |  |
|  |  | **449** | **PDKYKFSIRGFDPVT** |  |
|  |  | **450** | **DKYKFSIRGFDPVTD** |  |
|  |  | **468** | **PTTYFYMNRRVPLTN** |  |
|  | **B** | **456** | **YLPDKYKYNIAGFDP** | **YKYNIAGFD** |
|  |  | **457** | **LPDKYKYNIAGFDPE** |  |
|  |  | **458** | **PDKYKYNIAGFDPET** |  |
|  |  | **675** | **DVNFKYSGTIPYSDG** |  |
|  | **C** | **420** | **TVPSYEIDISATQRR** | **YEIDISATQ** |
|  |  | **421** | **VPSYEIDISATQRRN** |  |
|  | **D** | **86** | **LTQFFDSIYKTVDRG** | **FDSIYKTVD** |
|  |  | **87** | **QFFDSIYKTVDRGQR** |  |
|  |  | **88** | **FFDSIYKTVDRGQRN** |  |
|  |  | **90** | **DSIYKTVDRGQRNFE** |  |
|  |  | **239** | **TLNYLLQNRQSVPDT** |  |
|  |  | **410** | **SEAGWLADHRLPQAF** |  |
|  |  | **411** | **EAGWLADHRLPQAFD** |  |
|  | **E** | **427** | **SIPSYEIDLAASQRR** | **YEIDLAASQ** |
|  |  | **428** | **IPSYEIDLAASQRRN** |  |
|  |  | **713** | **APNLFEIKRHVGIDS** |  |
| **FIBRE** | **A** | **112** | **NGIDLNIDPKTLVVD** | **LNIDPKTLV** |
|  |  | **113** | **GIDLNIDPKTLVVDG** |  |
|  |  | **222** | **PTGNFVSSSNNPFNG** |  |
|  |  | **223** | **TGNFVSSSNNPFNGS** |  |
|  | **B** | **392** | **ITSLYIKLDRSQLEN** | **VKIDPAGPL** |
|  |  | **158** | **WELGVKIDPAGPLDA** |  |
|  |  | **159** | **ELGVKIDPAGPLDAS** |  |
|  | **C** | **392** | **ITSLYIKLDRSQLEN** | **VKPDPAGPL** |
|  |  | **306** | **TVSVKPDPAGPLTAS** |  |
|  | **D** | **142** | **GLDIAVDPSTLEVDD** | **IEVENKSLA** |
|  |  | **99** | **TTAPIEVENKSLALA** |  |
|  |  | **100** | **TAPIEVENKSLALAY** |  |
|  | **E** | **100** | **TAAPITVANKALTLA** | **ITVANKALT** |
|  |  | **101** | **AAPITVANKALTLAY** |  |

Supplementary Table 4: B-cell binding epitopes

| **Protein** | **Species** | **Sequence** | **Score** |
| --- | --- | --- | --- |
| PENTON | A | EILKQAPPMNVSSVCD | 0.94 |
|  | B | TETIQLDNRSCWGGDL | 0.93 |
|  | C | ASDIDTYNKDANHSNF | 0.94 |
|  | D | GIGAMYTSMPDTFVAP | 0.92 |
|  | E | SVPPAPPSSPVSGVPP | 0.94 |
| HEXON | A | RFHIQVPQKYFAIKNL | 0.94 |
|  | B | AIASMSGTVPNPNLGP | 0.97 |
|  | C | AGEGYGPDLSQIKLYT | 0.95 |
|  | D | TGPARAYRTPRVGGMS | 0.94 |
|  | E | TQTDDTPNSYRVRYSL | 0.94 |
| FIBRE | A | DPDAEAPTGKMARAGP | 0.94 |
|  | B | TVTWSKFLPDVNYTNP | 0.94 |
|  | C | TVTWSKFLPDVNYTNP | 0.94 |
|  | D | EGSIQSSNAGLAVKTD | 0.96 |
|  | E | GSTGGGGGGGGSGGNP | 0.97 |

Supplementary Table 5: List of Discontinuous B-Cell epitopes

| **S.no** | **Discontinuous residues** | **Number of residues** | **Score** |
| --- | --- | --- | --- |
| 1 | A:A38, A:D39, A:A40, A:K41, A:P42, A:L43, A:A44, A:T47, A:M54, A:A57, A:Y58, A:E60, A:A61, A:L62, A:F63, A:N64, A:N65, A:M66, A:L67, A:A68, A:A69, A:Y70, A:T71, A:E72, A:Y73, A:L74, A:G75, A:A76, A:V77, A:A78, A:V79, A:A80, A:A81, A:Y82, A:R83, A:E84, A:I85, A:A86, A:N87, A:A88, A:S89, A:L90, A:L91, A:A92, A:A93, A:Y94, A:E96, A:A97, A:F98, A:F99, A:N100, A:N101, A:I103, A:A104, A:A105, A:Y106, A:G107, A:E108, A:L109, A:D110, A:L111, A:V112, A:Y113, A:P114, A:F115, A:A116, A:A117, A:Y118, A:H119, A:E120 | 70 | 0.756 |
| 2 | A:F149, A:A152, A:A153, A:L155, A:E156, A:L157, A:T158, A:N159, A:Q162, A:L163, A:K164, A:K165, A:V166, A:V167, A:A168, A:D169, A:A170, A:R171, A:P172, A:L173, A:L174, A:K175, A:K176, A:F177, A:Y178 | 25 | 0.721 |
| 3 | A:V20, A:A21, A:A22, A:Y23, A:I24, A:E25, A:L26, A:D27, A:N28, A:A29, A:A30, A:P31, A:L32, A:A33, A:E36, A:L37, A:A141, A:Y142, A:A143, A:E144, A:N145, A:S146, A:S147, A:V148, A:K150, A:F151, A:D191, A:K193, A:A194, A:S195, A:D196, A:K197, A:K198, A:F199, A:V200, A:P201, A:V202, A:Q203, A:R204 | 39 | 0.716 |
| 4 | A:L316, A:E317, A:K318, A:K319, A:I320, A:T321, A:V322, A:A323, A:N324, A:K325, A:A326, A:L327, A:T328, A:G329, A:P330, A:G331, A:P332, A:G333, A:E334, A:I335, A:Q338, A:D349, A:G350, A:P351, A:G352, A:P353, A:G354, A:A355, A:I356, A:A357, A:S358, A:M359, A:S360, A:G361, A:T362, A:V363, A:P364, A:N365, A:P366, A:N367, A:L368, A:G369, A:P370, A:G371, A:P372, A:G373, A:P374, A:G375, A:A376, A:G377, A:E378, A:G379, A:Y380, A:G381, A:P382, A:D383, A:L384, A:S385, A:Q386, A:I387, A:K388, A:L389, A:Y390, A:T391, A:G392, A:P393, A:G394, A:P395, A:G396, A:E397, A:G398, A:S399, A:I400, A:Q401, A:S402 | 75 | 0.704 |
| 5 | A:G428, A:S429, A:G430, A:G431, A:N432, A:P433 | 6 | 0.599 |
| 6 | A:I238, A:P239, A:Y240 | 3 | 0.5 |

Supplementary Figures:


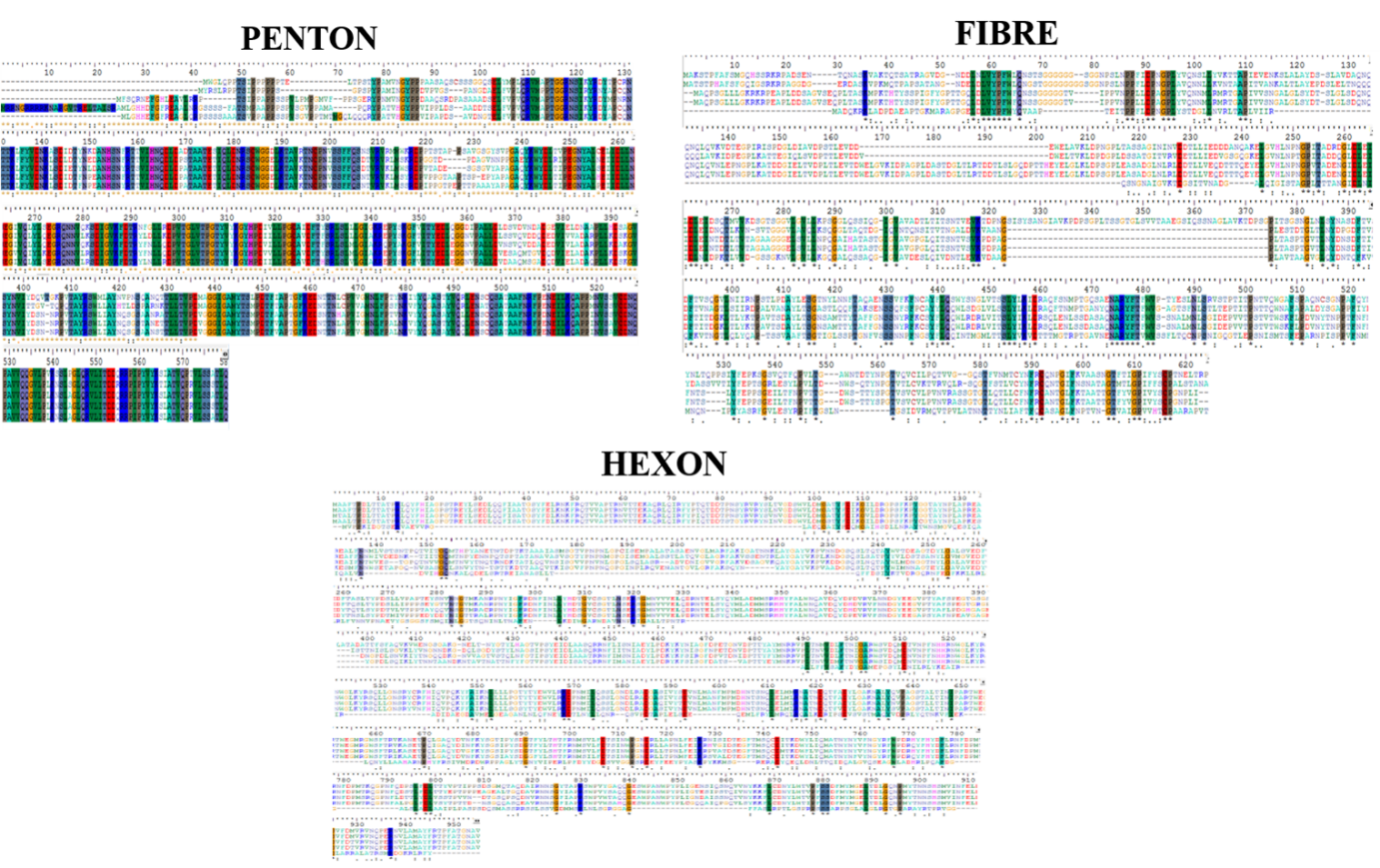


Supplementary Figure 1: This figure displays a multiple sequence alignment (MSA) of three key proteins essential to the structure and function of adenoviruses: penton, fiber, and hexon. The MSA illustrates the conservation of amino acid sequences across various adenovirus strains or serotypes. Each row in the alignment represents a different strain or serotype, while columns represent individual amino acid positions within the proteins


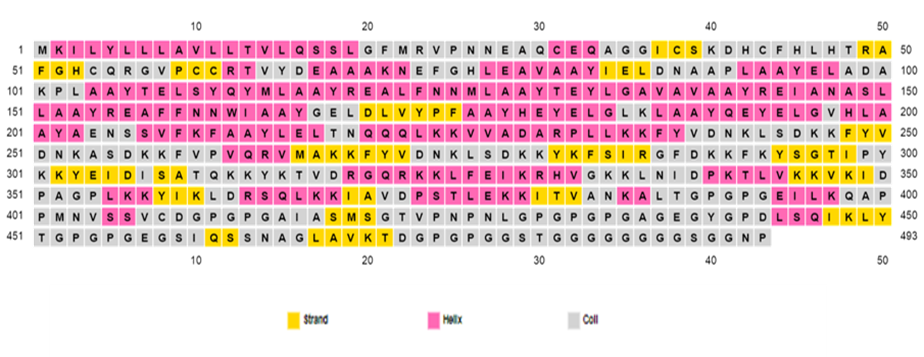
 Supplementary Figure 2: This figure depicts the predicted secondary structure of the designed multi-epitopic vaccine against FAdV. The secondary structure elements, including alpha helices, beta strands, and coils, are annotated based on computational modeling and bioinformatics analysis.


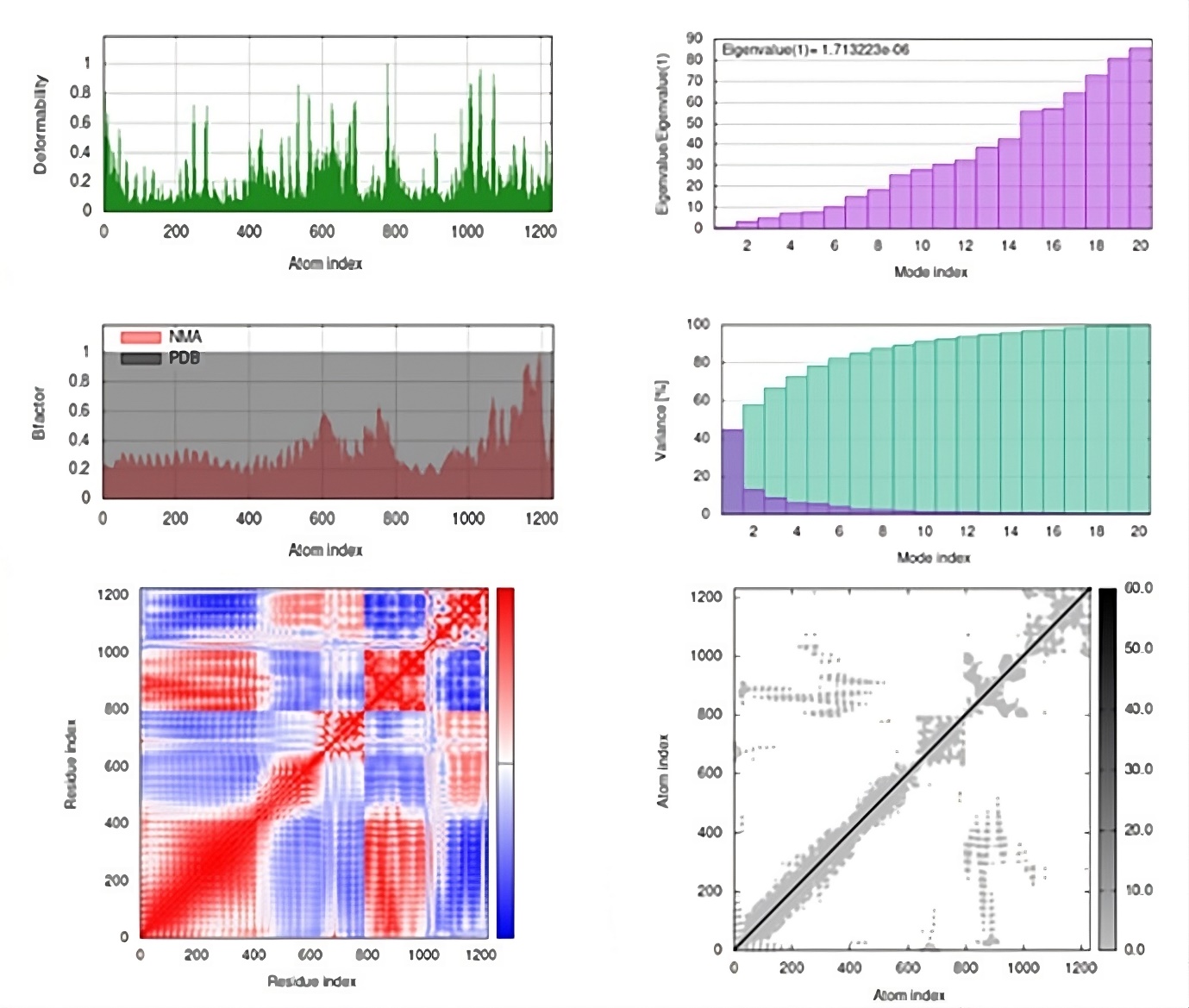
 Supplementary Figure 3: This figure presents a comprehensive Normal Mode Analysis (NMA) of a docked protein complex (TLR2 and multi-epitopic vaccine), offering insights into its dynamic behavior and structural flexibility through various analytical methods.


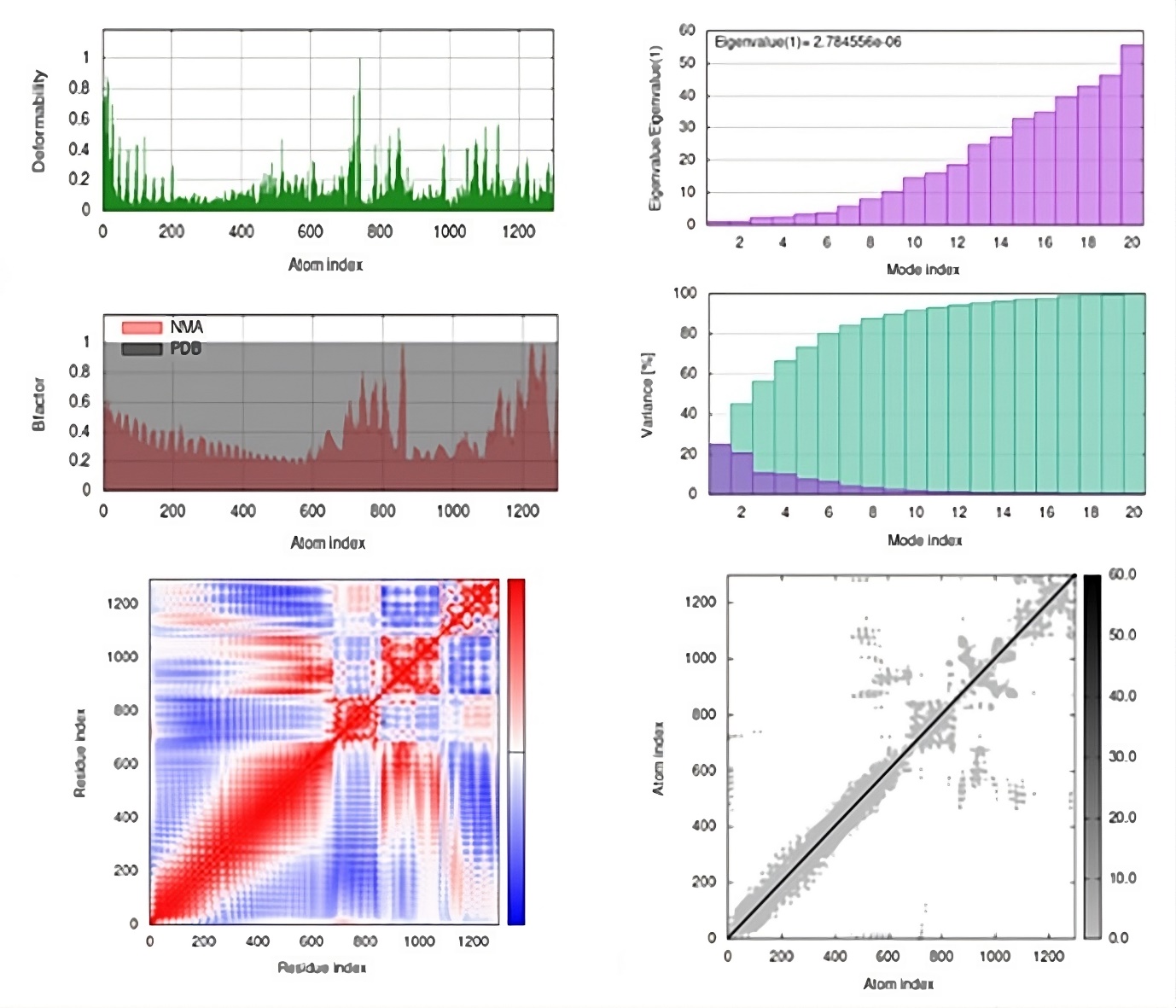


Supplementary Figure 4: This figure presents a comprehensive Normal Mode Analysis (NMA) of a docked protein complex (TLR5 and multi-epitopic vaccine), offering insights into its dynamic behavior and structural flexibility through various analytical methods.
